# A Stress-Induced Digital Escapism Framework for Understanding the Link Between Stress and Problematic Social Media Use

**DOI:** 10.3390/bs16060853

**Published:** 2026-05-26

**Authors:** Hwajin Yang, Frosch Y. X. Quek, Salin X. H. Yap, Germaine Y. Q. Tng, Gilaine Rui Ng

**Affiliations:** 1 School of Social Sciences, Singapore Management University, 10 Caning Rise, Singapore 179873, Singapore; yixuan.quek.2021@phdps.smu.edu.sg (F.Y.X.Q.); salin.yap.2020@business.smu.edu.sg (S.X.H.Y.); gilaineng@smu.edu.sg (G.R.N.); 2 School of Social Sciences, Nanyang Technological University, 50 Nanyang Ave., Singapore 639798, Singapore; germaine.tngqi@ntu.edu.sg

**Keywords:** stress-induced digital escapism framework, external stress demands, problematic social media use, internal stress reactions, maladaptive emotion regulation strategies, escapism motives

## Abstract

Given that stress is a significant risk factor for problematic social media use, understanding the underlying psychological mechanisms is essential. We introduce the Stress-Induced Digital Escapism (SIDE) framework, which posits that negative internal emotional responses to external stressors may increase reliance on maladaptive emotion regulation strategies that fail to alleviate distress. These processes may, in turn, strengthen escapism motives that lead to seeking emotional relief through compulsive social media use. Using structural equation modeling (*N* = 238), we examined three integrated psychological pathways—negative stress reactions, maladaptive emotion regulation strategies, and escapism motives—as sequential mediators linking external demands to problematic social media use. Consistent with the proposed framework, external demands showed significant indirect associations with problematic social media use through negative emotional responses, maladaptive interpersonal emotion regulation strategies (venting, reassurance-seeking), and escapism motives as sequential mediators. Sensitivity analysis supported the robustness of the serial mediation model over alternative models with reversed pathways. These findings support the SIDE framework as a unified account of the psychological mechanisms underlying stress-related problematic social media use.

## 1. Introduction

Since their emergence in the early 2000s, social media platforms have rapidly evolved to serve a wide variety of functions: socializing and networking, managing a business, handling personal finances, and seeking information (Aichner et al., 2021). In addition to the multifunctionality of social media, its heightened focus on social interaction and external validation (e.g., likes, dislikes, shares, and comments)—as well as AI-driven algorithms that continuously deliver personalized content—further intensifies habitual engagement with social media (Pellegrino et al., 2022). Moreover, the ease of forming extensive social networks and the growing popularity of short-form videos across various social media platforms (e.g., YouTube Shorts, TikTok, Instagram Reels) have contributed to more habitual and potentially problematic social media use. Not surprisingly, research on problematic social media use has documented notable adverse outcomes, including lower self-esteem (Khoo & Yang, 2024), increased loneliness and depression (Wu et al., 2024; Yigiter et al., 2024), and poorer body image among youths (Yang et al., 2020). Beyond these established links, recent evidence from diverse student populations further underscores the complex nexus between social media engagement and broader mental health outcomes (Hoque et al., 2026).

Given these concerning outcomes of problematic social media use, numerous studies have focused on understanding the risk factors of social media addiction, such as negative affect (Schivinski et al., 2020) and fear of missing out (Savci et al., 2022). In particular, stressors—defined as external demands that exceed an individual’s capacity and resources—have emerged as a significant factor that contributes to problematic social media use (Ng & Yang, 2026; Song & Park, 2019; Wang et al., 2021). When experiencing stressors such as workplace pressures, social isolation, interpersonal difficulties, or personal problems, individuals may turn to social media for immediate distraction or stress relief. In line with this notion, studies suggest that stress contributes to the onset and development of problematic social media use (e.g., Wolfers & Utz, 2022); smartphone addiction (e.g., Samaha & Hawi, 2016); and internet addiction (e.g., Song & Park, 2019; Tavolacci et al., 2013).

Despite the well-documented link between stress and problematic social media use, relatively few studies have examined the psychological mechanisms underlying this association. Previous research has primarily focused on either the motivational need to seek social support online (e.g., Brailovskaia et al., 2021) or the need to cope with negative emotions (Zhu et al., 2025). However, these pathways have largely been examined in isolation, and less is known about how motivational, emotional, and regulatory processes jointly contribute to stress-related problematic social media use. Specifically, studies examining anxiety and low self-esteem as mediators (Zhu et al., 2025; Hou et al., 2019; Khoo & Yang, 2024) have often overlooked the reactive regulatory strategies people use to manage emotional distress. Conversely, studies identifying motivational factors, such as escapism, as predictors of problematic social media use (Süral et al., 2019) have rarely considered the emotional triggers and unsuccessful regulation that may reinforce these motives. For instance, individuals may turn to social media for escape when they lack effective regulation strategies or when their regulatory attempts to manage negative emotions are unsuccessful. Together, these gaps highlight the need for a more comprehensive framework that integrates emotional, regulatory, and motivational processes to explain stress-related problematic social media use.

### 1.1. Theoretical Positioning of the SIDE Framework

We introduce the Stress-Induced Digital Escapism (SIDE) framework to explain how external stress demands become linked to problematic social media use. The framework draws on existing theories of stress and coping, media motivation, compensatory internet use, and addictive internet use, but addresses a more specific question: how stress-related emotional distress becomes connected to maladaptive regulation and escapism-driven social media use.

The SIDE framework is grounded in the Transactional Model of Stress and Coping, which conceptualizes stress as a dynamic process involving appraisal and coping (Lazarus & Folkman, 1984). From this perspective, external demands become stressful when individuals appraise them as taxing or exceeding their available resources, thereby prompting coping efforts to manage the situation or the emotional distress it elicits. This transactional model provides a foundation for SIDE by framing stress as a process shaped by appraisal and coping, rather than as a direct cause of problematic behavior. However, it does not fully explain how maladaptive coping with stress-related distress may become channeled into digital media use.

SIDE also draws on Uses and Gratifications Theory (UGT), which proposes that media users actively select media to satisfy psychological and social needs (Katz et al., 1973). In the context of social media, these needs may include entertainment, social connection, mood management, information seeking, self-presentation, and escapism. UGT helps explain why individuals may turn to social media when distressed, particularly when they expect it to provide distraction, emotional relief, or social validation. However, because UGT primarily emphasizes motives and gratifications, it does not explain how stress-related emotional distress and ineffective regulation may give rise to escapism motives.

The SIDE framework further builds on, rather than replaces, the Compensatory Internet Use Theory (CIUT). CIUT proposes that individuals turn to the internet to cope with negative life situations, psychosocial problems, or unmet needs (Kardefelt-Winther, 2014). This account is valuable because it shifts attention from the technology itself to the psychological contexts and motives underlying excessive use. However, CIUT provides a broad compensatory explanation and does not specify in detail how stress-related emotional responses and maladaptive regulation processes contribute to escapism-driven problematic social media use.

The SIDE framework also complements the Interaction of Person-Affect-Cognition-Execution (I-PACE) model, a broad process model of specific internet-use disorders (Brand et al., 2016). The I-PACE model proposes that predisposing characteristics, affective and cognitive responses to internal or external triggers, expectations of relief or reward from internet use, coping styles, craving, and executive control processes interact in the development and maintenance of problematic internet use. Its strength lies in integrating multiple person-level and situational factors into a comprehensive account of addictive internet-use behaviors. However, because of its broad scope, the I-PACE model provides only limited specificity in explaining how stressors are translated into escapism-driven problematic social media use. Although it recognizes affective responses, coping processes, and self-regulatory control, it does not specify how these processes may unfold sequentially from stress-related distress to escapism-driven use. Taken together, these theories provide important foundations for understanding stress, coping, media motives, and problematic internet use. The SIDE framework integrates these insights into a targeted account of stress-related problematic social media use by specifying a sequential pathway from external stress demands to negative emotional responses, maladaptive emotion regulation, escapism motives, and problematic use.

### 1.2. Stress-Induced Digital Escapism (SIDE) Framework

**Figure 1:**
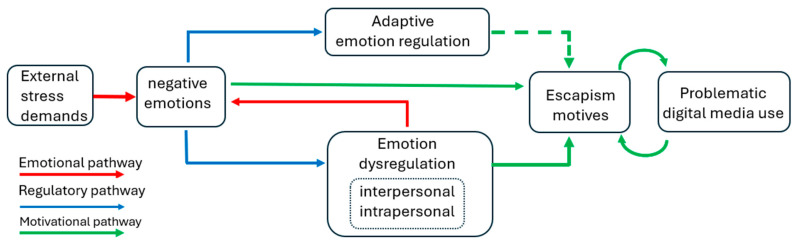
A Stress-Induced Digital Escapism (SIDE) framework for problematic social media use.

As illustrated in Figure 1, the SIDE framework proposes three connected pathways linking external stress demands to problematic social media use: emotional, regulatory, and motivational. The emotional pathway begins with external stress demands that elicit negative emotional responses. The regulatory pathway explains how individuals respond to these emotions through adaptive or maladaptive regulation strategies. The motivational pathway explains how unresolved emotional distress and maladaptive regulation may strengthen escapism motives, increasing the likelihood that social media use becomes a tool for emotional relief. This section outlines each pathway in turn.

***The emotional pathway***. The emotional pathway begins with external stress demands and the negative emotional responses they elicit. Consistent with stress and coping theory, stressful demands are likely to generate distress when individuals appraise them as taxing or exceeding their coping resources (Lazarus & Folkman, 1984). Empirical work also links stressors with negative affective states, including worry, tension, anxiety, and reduced well-being (Brougham et al., 2009; P. Y. Chen & Spector, 1991). Within the SIDE framework, these negative emotional responses are important because they create the need for regulation. Thus, the model does not assume a direct path from stress to problematic use; rather, it begins with stress-related emotional distress and examines how individuals respond to those emotions.

***The regulatory pathway***. Once negative emotions are activated, individuals may rely on either adaptive or maladaptive regulation strategies. Adaptive strategies may help reduce distress and lower the likelihood of turning to social media for emotional relief. In contrast, maladaptive strategies may provide short-term relief while leaving the underlying distress unresolved. In the present study, we focus on three maladaptive strategies: avoidance, venting, and reassurance-seeking. Avoidance involves disengaging from distressing situations or emotional discomfort; venting involves expressing negative emotions to others; and reassurance-seeking involves repeatedly seeking validation or comfort from others to reduce uncertainty or distress (Abe & Nakashima, 2022; Aldao et al., 2010).

Although these strategies may be understandable responses to stress, repeated reliance on them can maintain or intensify negative emotions. Avoidance may reduce immediate discomfort but prevent direct engagement with the source of distress. Venting may provide momentary relief but can sustain attention to negative emotions when used repeatedly. Reassurance-seeking may temporarily reduce uncertainty but can foster dependence on external validation. Consistent with broader emotion regulation research, such strategies are often associated with poorer emotional adjustment compared with more adaptive forms of regulation (Aldao et al., 2010; Gross & John, 2003). In the SIDE framework, maladaptive regulation therefore serves as a bridge between stress-related negative emotions and the motivation to escape those emotions through social media.

***The motivational pathway***. The motivational pathway explains how unresolved emotional distress may become linked to problematic social media use. When negative emotions persist despite regulatory efforts, individuals may become more motivated to use social media as a way to escape stress, discomfort, or unwanted internal states. Escapism motives are therefore positioned as a proximal mechanism linking maladaptive regulation to problematic social media use. Prior studies show that escapism is associated with problematic forms of online engagement, including excessive internet use, problematic Instagram use, and social network addiction (Cuadrado et al., 2022; Kırcaburun & Griffiths, 2019; Ohno, 2016).

Importantly, the SIDE framework also emphasizes a reinforcing process between escapism motives and problematic social media use. Social media may provide immediate distraction or emotional relief, which can strengthen the tendency to use it again when distress recurs. Over time, repeated reliance on social media for emotional relief may make use more habitual and harder to regulate. In this way, the SIDE framework integrates emotional distress, maladaptive regulation, and escapism motives into a unified account of stress-related problematic social media use.

### 1.3. The Current Study

Guided by the SIDE framework, the present study examined the psychological mechanisms underlying the link between stress and problematic social media use. Using a latent variable approach to reduce measurement error, we tested a serial mediation model in which negative emotional reactions, emotion regulation strategies, and escapism motives sequentially mediated the association between external stressors and problematic social media use. We examined three maladaptive regulation strategies, avoidance, venting, and reassurance-seeking, and one adaptive strategy, acceptance. We hypothesized that external stressors would be indirectly associated with problematic social media use through heightened negative emotional reactions, greater reliance on maladaptive emotion regulation strategies, and stronger escapism motives. Specifically, we expected avoidance, venting, and reassurance-seeking to strengthen the pathway from negative emotional reactions to escapism motives and, in turn, problematic social media use. In contrast, we expected acceptance to weaken or eliminate this pathway by reducing the likelihood that negative emotional reactions would develop into escapism motives.

## 2. Materials and Methods

### 2.1. Participants

Two hundred and fifty-eight undergraduate students from a university in Singapore were recruited over 2 months through the university’s research participation system. Participants were enrolled in psychology modules that offered research participation credit, and no specific inclusion or exclusion criteria were applied. The sample was predominantly female (81%; age range = 18–28 years; M = 21.42), which broadly reflects the sex distribution of students enrolled in these psychology modules rather than targeted recruitment by sex (see Table 1 for detailed descriptive statistics).

### 2.2. Measures

**Perceived stress.** We assessed participants’ perceived stress using the 20-item revised Perceived Stress Questionnaire (PSQ-20; Fliege et al., 2005). Participants rated on a four-point scale the frequency with which each item applied to them in the past month (1 = *almost never*, 2 = *sometimes*, 3 = *often*, and 4 = *usually*). The scale consisted of four subscales: demands (external stressors such as deadlines and time pressure); worries (anxious concern for the future and feelings of desperation and frustration); tension (disquietude, exhaustion, and lack of relaxation); joy (positive feelings such as joy and energy). Joy was reverse-coded to align with negative emotional responses associated with stress (i.e., lack of joy). According to the established psychometrics of the scale (Fliege et al., 2005), the demands subscale reflects perceived external stressors, while the three subscales—worries, tension, and lack of joy—represent internal stress reactions (Fliege et al., 2005). Perceived external stressors pertain to environmental demands that contribute to stress levels. In contrast, internal stress reactions denote subjective responses to stress as they manifest in an individual’s thoughts and emotions. Different stress dimensions were calculated separately by summing the relevant subscale scores, with higher scores indicating elevated levels of perceived stress demands and internal emotional reactions to stress.

**Emotion regulation strategies.** The Difficulties in Interpersonal Regulation of Emotions (DIRE) questionnaire is a scenario-based measure used to identify potentially maladaptive forms of emotion regulation (Dixon-Gordon et al., 2018). Participants read three emotionally evocative scenarios set in the context of work, friendship, and romantic relationships (e.g., “You are fighting with a significant other…”) and rated the likelihood (1 = *very unlikely* to 5 = *very likely*) that they would respond in a variety of ways. The seven items for each scenario reflected four emotion regulation strategies: venting (e.g., “Raise your voice or complain to the person in charge”); avoidance (e.g., “Distract yourself from how you are feeling”); reassurance-seeking (e.g., “Keep asking for reassurance”); and acceptance (e.g., “Simply notice your feelings”). Engagement in each emotion regulation strategy was indexed by the mean value of relevant items from corresponding scales across the three emotional scenarios.

**Escapism motives for social media use**. An adapted version of the Motivation for Using Instagram Scale was used to assess motives for social media use (E. Lee et al., 2015). To align the measure with the broader scope of the present study, which focused on social media use rather than Instagram use specifically, references to Instagram were replaced with social media. Participants rated 14 items assessing reasons for using social media on a 7-point scale (1 = *strongly disagree* to 7 = *strongly agree*). Consistent with the SIDE framework, we used the escapism subscale (three items; e.g., “To escape from reality,” “To forget about troubles,” and “To avoid loneliness”). The original measure also includes other motives such as social interaction (e.g., “To interact with a number of people”) and peeking (“To browse daily lives of celebrities or others”), but the present analyses focused on escapism. Escapism indices were created by averaging the corresponding items.

**Social media addiction**. Problematic social media use was assessed using the Bergen Social Media Addiction Scale (BSMAS; Andreassen et al., 2016). Participants responded to the 6-item scale regarding the frequency of social media use behaviors over the past year (e.g., “…felt an urge to use social media more and more”) on a 5-point scale (1 = *very rarely* to 5 = *very often*). The total score was calculated (ranging from 6 to 30), with higher scores indicating a higher tendency toward problematic social media use.

### 2.3. Covariates

Age, sex (0 = male, 1 = female), and subjective socioeconomic status (SES) were used as covariates in all analyses because of their relevance to stress, emotion regulation, and addictive behavior (Brougham et al., 2009; Zhao et al., 2022). Subjective SES was assessed using the MacArthur Scale of Subjective Social Status. Sex was included because female college students have been found to report higher stress and greater use of emotion-focused coping strategies than male students (Brougham et al., 2009). Recent work also highlights the relevance of sex differences in digital media research, further supporting their inclusion as a covariate (Olijo, 2025). Age was included because coping resources and regulatory capacities may vary across the life span (Lazarus, 1966).

### 2.4. Procedure

The survey was administered online using Qualtrics. Participants first responded to basic demographic questions (age, sex, and subjective SES). They then responded to questions relating to perceived stress, emotion regulation, motivations for social media use, and social media addiction. Upon completing the survey, participants received either course credit or $10 in cash. The study’s procedure was approved by the university’s Institutional Review Board (IRB approval number: IRB-21-181-A132(1121)), and all procedures were conducted in accordance with the Declaration of Helsinki. Informed consent was obtained from all participants.

### 2.5. Analytic Plan

All analyses were conducted in R Studio using R version 4.4.1 (R Core Team, 2024). Packages used include “lavaan” version 0.6–18 (Rosseel, 2012); “semTools” version 0.5–6 (Jorgensen et al., 2022); “psych” version 2.4.6.26 (Revelle, 2024); and “Hmisc” version 5.1–3 (Harrell, 2024). Missing data were handled using full information maximum likelihood (FIML), which assumes that data are missing at random conditional on the variables included in the model. Missing values primarily reflected incomplete survey responses, with no design-based or substantive indication that missingness systematically depended on unobserved responses after accounting for model variables.

The latent construct of external demands (as a predictor) was modeled using the five subscale items from the PSQ-20 as indicators. The latent construct of internal stress reactions (as the first mediator)—which was initially modeled using the three subscale scores of worries, tension, and lack of joy as indicators—did not yield acceptable model fit. Therefore, internal stress reaction was modeled as a manifest variable based on the mean scores of the three subscales (worries, tension, and lack of joy). Venting, reassurance-seeking, avoidance, and acceptance strategies (as the second mediators) were modeled as latent variables, with each represented by three parcels as indicators; each parcel was calculated by the mean score of the corresponding items within each scenario. Parceling was used to improve model fit and the psychometric properties of the unidimensional constructs (Rhemtulla, 2016). The latent construct of escapism motive for social media use (as the third mediator) and problematic social media use (the outcome variable) were modeled using their corresponding scale items.

For all measurement models, confirmatory factor analysis was carried out to ensure that the indicators reflected their intended constructs. Thereafter, the model fit of the full measurement models for each emotion regulation strategy was tested. Finally, a series of structural equation modeling (SEM) analyses were performed to examine the hypothesized serial mediation process between stress (perceived external demands) and problematic social media use through internal stress reactions, emotion regulation strategies (acceptance, reassurance-seeking, venting, and avoidance), and escapism motive as serial mediators. We estimated separate SEMs for each emotion regulation strategy rather than entering all strategies into a single model. This approach was chosen because the primary aim was to test whether each theoretically distinct strategy supported the proposed SIDE sequence, rather than to compare their relative effects.

The model fit of each measurement model, full measurement models, and overall structural equation models was tested using the following criteria: Chi-square values, comparative fit index (CFI) ≥ 0.90 for acceptable fit; Tucker–Lewis index (TLI) ≥ 0.9 for good fit; root mean square error of approximation (RMSEA) ≤ 0.08 for acceptable fit; and standardized root mean square residual (SRMR) ≤ 0.08 for good fit. All reported estimates were standardized. In all SEM analyses, bootstrapping with 1000 resamples was used to fit the models and determine standard errors for each direct and indirect path parameter. Covariates for age, sex, and subjective SES were added to the adjusted SEM models to account for potential confounding effects.

## 3. Results

Descriptive statistics and zero-order correlational coefficients for all variables are reported in Table 1 and Table 2, respectively. Overall, both external demands and internal stress reactions demonstrated moderate correlations with the three maladaptive emotion regulation strategies—venting, avoidance, and reassurance-seeking—but showed no significant correlation with acceptance. Similarly, the escapism motive was moderately associated with the three maladaptive emotion regulation strategies but not with acceptance. Escapism motive was also strongly correlated with problematic social media use.

### 3.1. Measurement MODELS

Confirmatory factor analyses were conducted to evaluate the model fit of each measurement model. Individual measurement models for the latent constructs of external demands, acceptance, reassurance-seeking, venting, avoidance, escapism motive, and problematic social media use demonstrated good or acceptable model fit (see Table 3). To improve model fit for the latent constructs of problematic social media use (i.e., social media addiction) and escapism motive, we correlated similarly worded items within each scale in line with modification indices (e.g., for escapism motive, we correlated “To escape from reality” and “To forget about troubles”). All factor loadings were significant, which indicates that each indicator adequately represented the intended latent construct. All full measurement models were also found to have good or acceptable fit (see Table 3).

### 3.2. Structural Equation Modeling Analyses

In Model 1, external demands were specified as the predictor and problematic social media use as the outcome variable, with internal stress reactions, avoidance, and escapism motive included as serial mediators (see Table 4 for details). The indirect effect of external demands on problematic social media use via avoidance was not significant in both the unadjusted (γ = 0.042, SE = 0.034, 95% CI [−0.024, 0.108]) and adjusted model (γ = 0.042, SE = 0.049, 95% CI [−0.055, 0.139]; see Table 4 for details).

**Figure A1:**
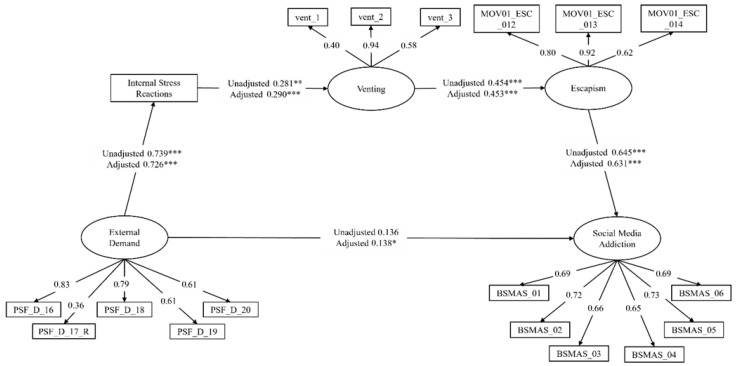
Serial mediation illustrating the relationship between stressors and problematic social media use (social media addiction) through internal stress reactions, venting, and escapism motive as serial mediators. * *p* < 0.05. ** *p* < 0.01. *** *p* < 0.001.

In Model 2, in which venting served as the second mediator (see Table 4 and Figure A1 in Appendix A), we found that the indirect effect of external demands on problematic social media use was significant (γ = 0.06, SE =0.030, 95% CI [0.002, 0.119]) in the unadjusted model. Similarly, the indirect effect was significant in the adjusted model (γ = 0.060, SE =0.028, 95% CI [0.006, 0.114]). These results confirmed our hypothesis.

**Figure A2:**
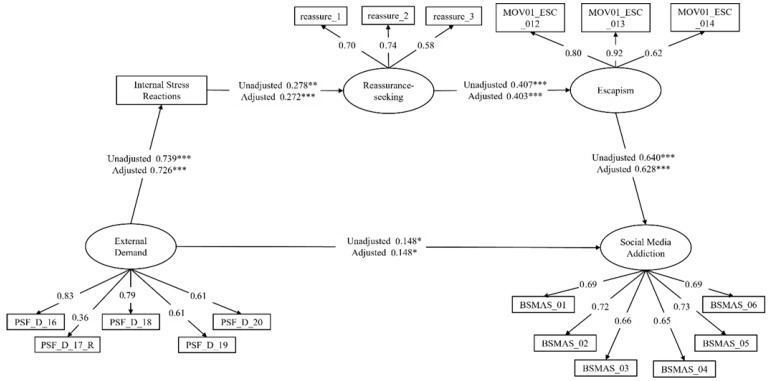
Serial mediation illustrating the relationship between stressors and problematic social media use (social media addiction) through internal stress reactions, reassurance-seeking, and escapism motive as serial mediators. * *p* < 0.05. ** *p* < 0.01. *** *p* < 0.001.

In Model 3, similar analyses were conducted with reassurance-seeking as the second mediator in place of venting. Consistent with the hypothesis, we found a significant indirect effect in both the unadjusted (γ = 0.054, SE =0.027, 95% CI [0.001, 0.106]) and adjusted model (γ = 0.050, SE = 0.025, 95% CI [0.001, 0.099]) when controlling for age, sex, and subjective SES (see Table 4 and Figure A2 in Appendix A).

In Model 4, with acceptance as the second mediator replacing maladaptive strategies, the indirect effect of external demands on problematic social media use via acceptance (as the 2nd mediator) was not significant, γ = 0.004, SE = 0.008, 95% CI [−0.011, 0.020], in the unadjusted model. Similar findings were found in the adjusted model with age, sex, and subjective SES controlled for (γ = 0.003, SE = 0.006, 95% CI [−0.009, 14]).

### 3.3. Sensitivity Analysis

To ensure the stability and robustness of our findings, we conducted a series of robustness checks by testing alternative models. These checks were conducted to compare the fit of the hypothesized models with plausible alternative specifications. Although such comparisons cannot establish causal directionality, they help evaluate whether the hypothesized models provide a better statistical account of the observed data than reversed-pathway alternatives. Specifically, we tested alternative models by reversing the direction of the proposed mediation pathway. In these models, problematic social media use was specified as the predictor variable, with escapism motives, each emotion regulation strategy, and internal stress reactions as serial mediators, and perceived external demands as the outcome variable. Model comparisons were conducted using changes in the comparative fit index (ΔCFI), along with the Akaike information criterion (AIC) and Bayesian information criterion (BIC). A ΔCFI of less than or equal to 0.01 was considered negligible, based on recommendations by Cheung and Rensvold (2002) and F. F. Chen (2007). Covariates such as age, sex, and subjective SES were included in all models to account for potential confounding effects. Fit indices for our hypothesized models and alternative models are presented in Table 5. All alternative models yielded a ΔCFI of less than 0.01, which suggests that the changes in model fit were negligible and that the alternative models did not improve fit relative to the hypothesized models.

## 4. Discussion

This study investigated the psychological mechanism that underlies the relationship between external stressors and problematic social media use (i.e., social media addiction). Supporting, in part, the Stress-Induced Digital Escapism (SIDE) framework, we identified significant indirect effects of external stressors on problematic social media use through the serial mediators of (a) negative emotional reactions, (b) increased use of maladaptive emotion regulation strategies (venting and reassurance-seeking), and (c) escapism motives for social media use. Our findings suggest that external stress demands that exceed perceived coping resources may be associated with heightened negative internal reactions. These reactions may, in turn, increase reliance on maladaptive regulation strategies that offer temporary relief from emotional discomfort (Messina et al., 2022). However, reliance on these maladaptive strategies may inadvertently reinforce escapism-related problematic social media use, because they do not effectively resolve emotional distress and underlying issues in the long run. Our findings provide partial support for the SIDE framework by identifying emotional, regulatory, and motivational pathways linking external stressors to problematic social media use.

Our findings highlight the link between maladaptive interpersonal emotion regulation strategies—venting and reassurance-seeking—and escapism motives associated with problematic social media use. Emotional venting, characterized by perseverative fixation on one’s negative emotions and negative self-focus (Compas et al., 2001; Connor-Smith & Flachsbart, 2007), exacerbates emotional distress rather than alleviating it (e.g., Klostermann et al., 2011; Liverant et al., 2004). Reassurance-seeking, while beneficial in moderation, can also become maladaptive when frequently used, because it creates a cycle of dependence on external validation for temporary relief from negative emotions. Importantly, both strategies may fail to address underlying emotional difficulties while also straining social support networks, potentially increasing frustration, fatigue, or resentment for close others and reducing the availability of meaningful social support when it is most needed. This further undermines individuals’ capacity to develop adaptive regulation strategies and internal emotional resilience in the long run. Since these maladaptive strategies tend to perpetuate psychological distress, they may strengthen escapism motives and increase reliance on prolonged and habitual social media use as a means of escaping their emotional discomfort (Kardefelt-Winther, 2014). This reliance may exacerbate negative emotions and reinforce a harmful cycle of social media dependency, thereby further entrenching problematic social media use.

These findings extend the literature by underscoring how maladaptive regulation strategies contribute to escapism motives associated with problematic social media use. Specifically, venting and reassurance-seeking likely capitalize on the features of social media—such as public posts, status updates, and immediate feedback—that make these platforms particularly appealing for emotional externalization and validation. This aligns with prior research that suggests that social media amplifies maladaptive emotion regulation by reinforcing cycles of emotional dependence and escapism (Marino et al., 2018; Kardefelt-Winther, 2014). Our findings also extend prior work linking escapism motives to problematic digital engagement such as excessive gaming, problematic internet use, and social media addiction during the COVID-19 pandemic (Cuadrado et al., 2022; Jouhki et al., 2022; Ohno, 2016).

Notably, however, avoidance was not significantly associated with escapism motives for problematic social media use, although it was hypothesized to operate similarly to venting and reassurance-seeking by maintaining emotional distress and strengthening escapism motives. One possible explanation concerns the specific form of avoidance assessed in the present study. The avoidance subscale of the DIRE primarily captures a behavioral tendency to disengage from externally distressing situations. This differs from experiential avoidance in Acceptance and Commitment Therapy, which refers to efforts to avoid, suppress, or alter unwanted internal experiences, such as thoughts, emotions, memories, and bodily sensations, and encompasses a broader range of cognitive and emotional suppression processes (Hayes et al., 1996; Kashdan et al., 2006). This distinction may help explain the non-significant pathway observed in the present model. Experiential avoidance may be more closely linked to escapism motives because it involves attempts to escape from distressing internal experiences. Behavioral avoidance, by contrast, involves disengagement from external stressors and may therefore dampen emotional intensity or reduce the urgency to seek additional emotional relief through social media. Unlike venting and reassurance-seeking, which involve active emotional expression and interpersonal engagement, behavioral avoidance may be less closely aligned with the ways social media platforms are used for immediate emotional relief and social feedback. Thus, the null finding for avoidance may reflect the specific form of avoidance captured by the DIRE, rather than a true absence of association between avoidance-related processes and escapism motives.

In addition, the relatively modest reliability of the avoidance subscale (α = 0.67) may have attenuated the observed association between avoidance and escapism motives. The study may also have had limited power to detect this specific pathway, particularly if the true effect is small. Therefore, the non-significant finding should be interpreted cautiously, as it may partly reflect measurement limitations or limited statistical power rather than the absence of a meaningful association. Future research using more reliable and comprehensive measures, particularly measures that distinguish behavioral avoidance from experiential avoidance, would help clarify whether avoidance-related processes operate within the SIDE framework.

The study has several limitations that should be addressed in future research. First, the cross-sectional design precludes causal inferences about the proposed pathways linking external stressors to problematic social media use through internal reactions, maladaptive emotion regulation, and escapism motives. While stressors were modeled as predictors of problematic social media use, excessive and problematic social media use may also contribute to increased stress (Wolfers & Utz, 2022). To examine this possibility, we tested alternative serial mediation models in which problematic social media use was specified as the predictor and perceived external stressors as the outcome. However, these models did not show better model fit than our hypothesized models, which is consistent with the proposed direction from stressors to problematic social media use. Nevertheless, these sensitivity analyses do not establish temporal ordering or causality. Future research should therefore use longitudinal cross-lagged designs to examine the temporal sequence of these pathways and daily diary or experience-sampling approaches to capture within-person fluctuations in stress, emotion regulation, escapism motives, and problematic social media use over time.

Second, our self-report measure of problematic social media use is subject to measurement error, including social desirability and recall bias (Althubaiti, 2016). To address this limitation, future studies should incorporate objective measures, such as daily social media screen time, frequency of social media pickups, or notification checks, to more accurately index social media use (e.g., Coyne et al., 2023). Such measures would provide a useful complement to self-report data by capturing behavioral indicators of social media engagement. However, objective measures are not without limitations. Built-in tracking systems may vary in accuracy across operating systems and devices, and usage metrics alone cannot capture the psychological motives underlying social media use. In particular, they do not indicate whether social media use reflects escapism, habit, social connection, or more purposeful goals. This distinction is especially important for the SIDE framework, which emphasizes escapism motives as a key pathway linking stress-related experiences to problematic social media use. Future research should therefore combine objective indicators with general self-report measures and motivation-based assessments to provide a more comprehensive understanding of stress-related problematic social media use.

Third, our sample consisted of undergraduate students from a local university, which limits the generalizability of the findings. This group is likely to experience context-specific stressors, particularly those related to academic demands, which may differ from the stressors encountered by working adults or older populations. In the Singaporean university context, academic demands may be especially salient and may have strengthened the associations between perceived external stressors and emotion regulation processes. Such differences may, in turn, influence the strength and configuration of the pathways proposed in the SIDE framework. In addition, the predominantly female sample may further limit the generalizability of the findings to male students. The relatively small number of male participants limited our ability to examine whether the SIDE pathways operate similarly among male and female students. Future studies should replicate these findings in community samples with broader age ranges and more sex balanced representation, given that stressors, emotion regulation strategies, and motivations for social media use may differ by age and sex (Brummer et al., 2013; Stefaniak et al., 2022; Xu, 2022).

Examining diverse cultural groups is also important since cultural values and norms can influence stress perceptions and coping mechanisms (H. Lee et al., 2023). Singapore is often characterized as a relatively collectivistic context in which relational interdependence and social harmony are emphasized (Hofstede, 1980). In such contexts, interpersonal emotion regulation strategies, such as venting and reassurance-seeking, may be especially salient. This cultural context may partly explain why venting and reassurance-seeking, but not avoidance, emerged as significant mediators in the present study. Consistent with this interpretation, cross-cultural research indicates that cultural values shape emotion expression and the social consequences of emotion regulation (Butler et al., 2007; Matsumoto et al., 2008). Expanding the study to include more diverse cultural contexts will not only strengthen the external validity of the SIDE framework but also provide a more comprehensive understanding of the relationship between stressors, stress reactions, maladaptive emotion regulation strategies, and escapism motives for social media use.

Fourth, the present study focused on individual-level psychological pathways and did not directly examine the broader algorithmic environment in which social media use occurs. AI-driven personalized feeds may repeatedly present emotionally salient and personally relevant content, making social media a more immediate and self-sustaining vehicle for emotional escape. Such algorithmic environments may strengthen escapism-related pathways, particularly among individuals who rely on maladaptive emotion regulation strategies. Future research should examine how algorithmic features interact with psychological vulnerabilities using designs that capture these processes more directly, such as experiments that manipulate feed characteristics or intensive longitudinal studies assessing real-time emotional states and exposure to personalized content. Such work represents an important direction for extending the SIDE framework.

Finally, while the present study examined acceptance as an adaptive emotion regulation strategy, future research should examine additional protective and resilience factors that may disrupt the pathways proposed in the SIDE framework. Cognitive reappraisal, which involves reframing stressful situations in a more constructive light, has been consistently associated with reduced emotional distress and lower reliance on maladaptive coping strategies (Gross & John, 2003), and may therefore weaken the association between internal stress reactions and escapism motives. Similarly, mindfulness, characterized by non-judgmental awareness of present moment internal experiences, may reduce the perceived urgency to escape negative emotions through social media (Martínez-Rubio et al., 2023). Perceived social support represents another promising protective factor, given its well-documented role in buffering against stress-induced negative emotional reactions (Taylor et al., 2000). Identifying the conditions under which such protective factors disrupt the stress-to-addiction pathway would extend the theoretical scope of the SIDE framework and inform the development of targeted intervention strategies.

## 5. Conclusions

Social media, increasingly shaped by AI-driven algorithms, has become central to productivity, social connection, and entertainment. However, its immersive and personalized features may also contribute to emotional distress and problematic patterns of use, particularly among adolescents and young adults. Among the risk factors associated with problematic social media use, stressors are especially important because they may increase the tendency to use social media for emotional escape. To explain this process, we proposed the SIDE framework, which links stress reactions, maladaptive emotion regulation, and escapism motives to problematic social media use. Consistent with this framework, our findings suggest that negative emotional reactions to external stressors are associated with maladaptive emotion regulation strategies, which may in turn strengthen escapism motives. By clarifying these psychological pathways, the present study highlights two key targets for prevention and early intervention in nonclinical and subclinical populations: reducing maladaptive emotion regulation strategies used to cope with stress-related negative emotions and addressing escapism motives associated with using social media to avoid real-life stressors and emotional discomfort. These targets can be supported through cognitive-behavioral strategies that help users recognize stress-related triggers and adopt more adaptive coping responses, as well as digital literacy programs that increase awareness of habitual checking, algorithmically reinforced engagement, and escapism-driven use. In addition, contextual supports, such as parental monitoring or supervision, may help reduce the risk of negative well-being outcomes associated with problematic social media engagement, particularly among adolescents (Grund et al., 2025). Promoting healthier regulatory strategies, fewer escapism motives, and supportive social contexts may help reduce the risk of problematic social media use.

## Figures and Tables

**Table 1:** Descriptive statistics of predictors, covariates, and criterion variables.

	Mean	SD	Min	Max	Skewness	Kurtosis	Reliability ^a^
Age	21.42	1.74	18	28	0.66	0.33	-
Sex (female %) ^b^	0.81	0.40	0	1	−1.56	0.43	-
Subjective SES	5.92	1.41	1	10	−0.27	0.08	-
External demands	13.90	3.14	6	20	−0.19	−0.31	0.78
Internal stress reactions ^c^	40.04	8.24	19	60	−0.02	−0.22	0.91
Acceptance	3.34	0.98	1	5	−0.20	−0.41	0.77
Reassurance-seeking	2.79	0.95	1	5	0.03	−0.69	0.78
Venting	2.12	0.77	1	4.17	0.48	−0.63	0.70
Avoidance	3.15	0.80	1	5	−0.31	−0.06	0.67
Escapism motive	12.73	5.09	3	21	−0.28	−0.80	0.82
Problematic social media use	16.10	5.06	6	30	0.24	−0.21	0.84

*Note:* ^a^ Reliability estimates were computed based on Cronbach’s alpha. ^b^ Sex was coded as 1 = female, 0 = male. ^c^ Subscale scores for worries, tension, and lack of joy were computed to index internal stress reactions.

**Table 2:** Zero-order correlational coefficients for predictors, covariates, and criterion variables.

	1	2	3	4	5	6	7	8	9	10
1. Age										
2. Sex	−0.45 ***									
3. Subjective SES	−0.01	−0.08								
4. Internal stress reactions	−0.05	0.19 **	0.15 *							
5. External demands	−0.11	0.18 **	−0.04	0.70 ***						
6. Acceptance	0.16 **	−0.04	0.07	−0.05	0.03					
7. Reassurance-seeking	−0.08	0.12 *	0.04	0.19 **	0.18 **	0.04				
8. Venting	−0.06	0.07	0.11	0.23 ***	0.21 ***	0.03	0.51 ***			
9. Avoidance	0.13 *	0.14	−0.02	0.17 ***	0.18 ***	−0.01	−0.04	0.01		
10. Escapism motive	−0.15 *	0.08	−0.01	0.34 ***	0.29 ***	−0.11	0.20 ***	0.27 ***	0.23 ***	
11. Problematic social media use	−0.23 ***	0.15 *	0.03	0.24 ***	0.28 ***	−0.09	0.34 ***	0.35 ***	0.18 **	0.49 ***

Note. * *p* < 0.05. ** *p* < 0.01. *** *p* < 0.001.

**Table 3:** Model fit indices for measurement models and structural models.

Model	$\chi^{2}$	CFI	TLI	RMSEA	SRMR
**Measurement models**					
External demands	0.085	0.987	0.974	0.060	0.029
Acceptance	-	1.000	1.000	0.000	0.000
Reassurance-seeking	-	1.000	1.000	0.000	0.000
Venting	-	1.000	1.000	0.000	0.000
Avoidance	-	1.000	1.000	0.000	0.000
Escapism motive	-	1.000	1.000	0.000	0.000
Problematic social media use	0.155	0.992	0.986	0.044	0.023
**Full measurement models** **with**					
Acceptance	<0.001	0.962	0.953	0.045	0.055
Reassurance-seeking	0.002	0.968	0.961	0.041	0.050
Venting	<0.001	0.958	0.949	0.047	0.050
Avoidance	0.007	0.972	0.965	0.037	0.049
**Structural models**					
**Model 1** (avoidance as the 2nd mediator)					
Unadjusted	<0.001	0.926	0.913	0.060	0.084
Adjusted	<0.001	0.913	0.895	0.058	0.082
**Model 2** (venting as the 2nd mediator)					
Unadjusted	<0.001	0.912	0.896	0.067	0.083
Adjusted	<0.001	0.908	0.889	0.060	0.079
**Model 3** (reassurance-seeking as the 2nd mediator)					
Unadjusted	<0.001	0.922	0.907	0.063	0.086
Adjusted	<0.001	0.916	0.899	0.057	0.082
**Model 4** (acceptance as the 2nd mediator)					
Unadjusted	<0.001	0.916	0.900	0.066	0.099
Adjusted	<0.001	0.905	0.885	0.062	0.093

*Note:* Adjusted models include age, sex, and subjective SES as covariates.

**Table 4:** Standardized path coefficients of structural equation modeling with internal stress reactions, emotion regulation strategy, and escapism motive as serial mediators.

Models	Unadjusted			Adjusted		
	γ	SE	*p*	γ	SE	*p*
**Model 1**						
Demands 🡒 Internal stress reactions (ISRs)	0.739	0.047	<0.001	0.726	0.050	<0.001
ISR 🡒 *Avoidance*	0.263	0.097	0.007	0.234	0.093	0.012
*Avoidance* 🡒 Escapism	0.354	0.145	0.015	0.406	0.228	0.075
Escapism 🡒 Problematic social media use	0.616	0.074	<0.001	0.610	0.082	<0.001
**Model 2**						
Demands 🡒 ISRs	0.739	0.047	<0.001	0.726	0.051	<0.001
ISR 🡒 *Venting*	0.281	0.083	0.001	0.290	0.078	<0.001
*Venting* 🡒 Escapism	0.454	0.089	<0.001	0.453	0.093	<0.001
Escapism 🡒 Problematic social media use	0.645	0.080	<0.001	0.631	0.082	<0.001
**Model 3**						
Demands 🡒 ISRs	0.739	0.048	<0.001	0.726	0.050	<0.001
ISR 🡒 *Reassurance-seeking*	0.278	0.080	0.001	0.272	0.076	<0.001
*Reassurance-seeking* 🡒 Escapism	0.407	0.096	<0.001	0.403	0.097	<0.001
Escapism 🡒 Problematic social media use	0.640	0.083	<0.001	0.628	0.082	<0.001
**Model 4**						
Demands 🡒 ISRs	0.739	0.048	<0.001	0.726	0.052	<0.001
ISR 🡒 *Acceptance*	−0.063	0.088	0.475	−0.050	0.082	0.542
*Acceptance* 🡒 Escapism	−0.160	0.094	0.089	−0.125	0.098	0.199
Escapism 🡒 Problematic social media use	0.607	0.075	<0.001	0.597	0.075	<0.001

**Table 5:** Comparative model fit indices for hypothesized models and alternative models.

Model	CFI	ΔCFI	AIC	BIC
**Hypothesized models**				
Model 1 (avoidance as the 2nd mediator)	0.913		13,900	14,092
Model 2 (venting as the 2nd mediator)	0.908		13,752	13,943
Model 3 (RS as the 2nd mediator)	0.916		14,034	14,226
Model 4 (acceptance as the 2nd mediator)	0.905		13,974	14,166
**Alternative models**				
Model 1 (avoidance as the 2nd mediator)	0.909	0.004	13,907	14,099
Model 2 (venting as the 2nd mediator)	0.904	0.004	13,758	13,950
Model 3 (RS as the 2nd mediator)	0.912	0.004	14,041	14,232
Model 4 (acceptance as the 2nd mediator)	0.900	0.005	13,983	14,174

*Note:* Only adjusted models were used. RS = Reassurance-seeking.

## Data Availability

The datasets generated and/or analyzed during this current study are available in the Open Science Framework repository at https://doi.org/10.17605/OSF.IO/VH3UX.
